# Coupled Bayesian Identification of Residual Stress and Fracture Strength in Thin-Film Fragmentation: A Physics-Informed Neural Network Framework with Synthetic Validation of Interface Adhesion Energy

**DOI:** 10.3390/ma19132824

**Published:** 2026-07-02

**Authors:** Jun Li, Linan Li, Zhiyong Wang, Chuanwei Li, Shibin Wang, Kai Kang

**Affiliations:** 1 School of Mechanical Engineering, University of South China, 28 West Changsheng Road, Zhengxiang District, Hengyang 421001, China; 2 Department of Mechanics, School of Mechanical Engineering, Tianjin University, 135 Yaguan Road, Jinnan District, Tianjin 300350, China; 3 School of Mechanical Engineering, Tianjin University of Commerce, 409 Guangrong Road, Beichen District, Tianjin 300134, China

**Keywords:** thin films, residual stress, fracture strength, Bayesian inference, physics-informed neural networks, crack spacing, interface adhesion

## Abstract

Residual stress in brittle films on compliant substrates is routinely inferred from fragmentation experiments by combining an elastic stress-transfer model with a fracture strength criterion. This inversion is inherently coupled because the observed crack spacing depends jointly on the residual stress and the film fracture strength. Conventional closed-form estimators typically rely on a single feature, such as the cracking onset strain, and prescribe the fracture strength a priori, often at its bulk value. This practice discards most of the information encoded in the full crack-spacing evolution. It also obscures two sources of uncertainty: the intrinsic variability of thin-film fracture strength and the limited sensitivity of any single observable to individual parameters. Here, we recast the inversion as a Bayesian physics-informed neural network (B-PINN) in which the entire measured curve of the mean crack spacing versus applied strain is likely to occur. Stochastic gradient Langevin dynamics then sample the joint posterior of residual stress and fracture strength. A central finding is that crack-spacing data alone constrain only the difference between fracture strength and residual stress, confining the posterior to a one-dimensional manifold in parameter space and leaving each quantity individually unresolved. A single substrate curvature measurement, which, through the Stoney relation, depends on the residual stress but not on the fracture strength, provides the missing orthogonal constraint and collapses the posterior to a tight, well-resolved region. We further derive an identifiability condition under which buckle-wavelength observations serve as a third independent channel for recovering interface adhesion energy, and provide a synthetic proof-of-concept of this three-channel extension on DLC/Si and Mo/Si datasets; an experimental validation of the adhesion channel is identified as the natural next step but lies beyond the present scope. Requiring only standard fragmentation measurements and a single non-destructive curvature scan, the framework converts a point-estimate procedure into a posterior-quantified inverse method that makes explicit what can, and cannot, be learned from thin-film mechanics experiments.

## 1. Introduction

Residual stress is a primary factor governing cracking, delamination, and durability of brittle films deposited on compliant or ductile substrates [1]. Several techniques are commonly used to quantify it [2,3,4,5,6], including X-ray diffraction, wafer curvature, Raman spectroscopy, and nanoindentation. Each is subject to specific limitations associated with specimen crystallinity, geometry, instrumentation, or constitutive assumptions. Fragmentation experiments offer a comparatively undemanding alternative, requiring only uniaxial tensile loading and optical observation of the evolving crack pattern. Because such experiments do not measure stress directly, an elastic stress-transfer model is required to recover the residual stress from the observed crack spacing. The one-dimensional formulation of Hsueh and Wereszczak [7] and the two-dimensional extension that we developed in earlier work [8] have become standard tools for this purpose in thin-film systems where direct stress measurement is impractical.

In [8], we estimated the residual stress in Cr films of three different thicknesses on a polyimide substrate by coupling a two-dimensional elastic stress field with a brittle-fracture strength criterion. The resulting closed-form expression connects the applied strain at the onset of cracking, the mean fragment length, and the assumed film fracture strength to the residual stress. This yields compressive values that decrease in magnitude with increasing film thickness. While mechanically well-grounded, this earlier inversion is considerably more restrictive than the underlying experiment: it exploits only a single point on the measured crack-spacing curve, prescribes the film fracture strength a priori, and returns a point estimate without an associated posterior uncertainty.

The present study revisits that formulation using a Bayesian framework approach in which the entire measured crack-spacing–strain response enters the likelihood, and the residual stress and film fracture strength are inferred jointly. The stress-transfer equations of our earlier model serve as the forward model and are enforced through a physics-informed neural network [9,10]. Stochastic gradient Langevin dynamics [11] is used to sample the joint posterior. This formulation preserves the mechanics underlying our earlier closed-form estimator while making its implicit assumptions explicit. A central result is that the crack-spacing curve alone is insufficient to disentangle the residual stress from the fracture strength. The posterior instead concentrates along an elongated manifold in the two-parameter plane, of which any closed-form point estimate (including our earlier one) represents a single projection obtained by prescribing the fracture strength in advance. A single substrate curvature measurement, which, via the Stoney relation, depends on the residual stress but not on the fracture strength, provides the missing orthogonal constraint and allows both quantities to be recovered from data rather than imposed by assumption.

The remainder of this paper is organised as follows. Section 2 reviews the governing stress-transfer model and introduces the notation used throughout. Section 3 formulates the Bayesian inverse problem and establishes that our earlier closed-form estimator [8] emerges as a limiting case. Section 4 re-analyses the Cr/polyimide data of [8] and demonstrates how the curvature measurement resolves the parameter degeneracy. Section 5 derives the identifiability condition under which buckle-wavelength observations provide a third independent channel. Section 6 validates the multi-channel extension on synthetic systems in which the interface adhesion energy is additionally recovered. Section 7 discusses the scope and limitations of the framework, and Section 8 summarises the principal conclusions.

## 2. Stress-Transfer Model

**Figure 1:**
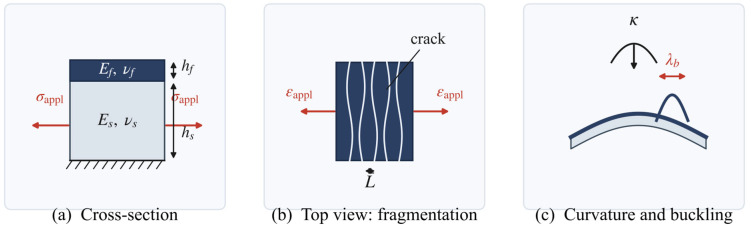
Schematic of the thin-film fragmentation experiment and the three observation channels. (**a**) Cross-sectional view of a film of Young’s modulus $E_{f}$, Poisson’s ratio $\nu_{f}$, and thickness $h_{f}$, perfectly bonded to a substrate of corresponding properties $E_{s}$, $\nu_{s}$, and $h_{s}$, subjected to remote uniaxial tension $\sigma_{\mathrm{appl}}$. (**b**) Top view of the fragmented film, showing the array of through-thickness cracks and the mean crack spacing $\bar{L}$ measured under applied substrate strain $\varepsilon_{\mathrm{appl}}$. (**c**) Out-of-plane response after unloading: substrate curvature κ probed by the Stoney relation, and steady-state buckle half-wavelength $\lambda_{b}$ where buckling is feasible.

We retain the plane strain film/substrate model developed in our previous work [8] and summarise it here in order to correct the notation. The geometry of the specimen and the three observation channels exploited in the present study are shown in Figure 1. A film of Young’s modulus $E_{f}$, Poisson’s ratio $\nu_{f}$, and thickness $h_{f}$ is perfectly bonded to a substrate of corresponding properties $E_{s}$, $\nu_{s}$, and $h_{s}$. The substrate is subjected to remote uniaxial tension, producing an applied stress $\sigma_{\mathrm{appl}}$ in the bonded region (equivalently, an applied strain $\varepsilon_{\mathrm{appl}}$). The pre-existing residual stress $\sigma_{0}$ in the film is represented by a uniform residual eigenstrain $\varepsilon_{\mathrm{res}}$, as follows:

(1) $$\varepsilon_{res}=-\frac{\left(1-\nu_{f}^{2}\right)\sigma_{0}}{E_{f}}$$

The analytical solution of Yin and Li [12,13] then yields the maximum tensile stress that is carried by the film within a fragment of mean length $\bar{L}$,

(2) $$\sigma_{x,max}^{f}=-\;\frac{\sigma_{appl}-\frac{E_{s}}{\left(1+\nu_{s})(1-\nu_{s}\right)}\varepsilon_{res}}{\frac{h_{f}}{h_{s}}+\frac{E_{s}}{E_{f}}\frac{\left(1+\nu_{f})(1-\nu_{f}\right)}{\left(1+\nu_{s})(1-\nu_{s}\right)}}\left[\frac{h_{f}d_{1}}{tan(d_{1}h_{f})cosh(2c_{1}\bar{L}/3)}-1\right]$$

where the geometric coefficients $c_{1}$ and $d_{1}$ are determined by the transcendental geometry equations we derived in [8] and depend only on the elastic constants and the film/substrate thickness ratio. Brittle fragmentation of the film is taken to occur when the maximum tensile stress in the film attains its fracture strength,

(3) $$\sigma_{str}=\sigma_{x,max}^{f}\left(\sigma_{0},\varepsilon_{appl},\bar{L}\right)$$

Equation (3) constitutes the observation equation of the inverse problem. For a single measured pair $\left(\bar{L},\;\varepsilon_{\mathrm{appl}}\right)$, one algebraic relationship between $\sigma_{0}$ and $\sigma_{\mathrm{str}}$ is provided. As such, a single observation is therefore insufficient to identify the two parameters separately, a point that motivates the identifiability analysis of Section 3.

## 3. Bayesian Inverse Formulation

**Figure 2:**
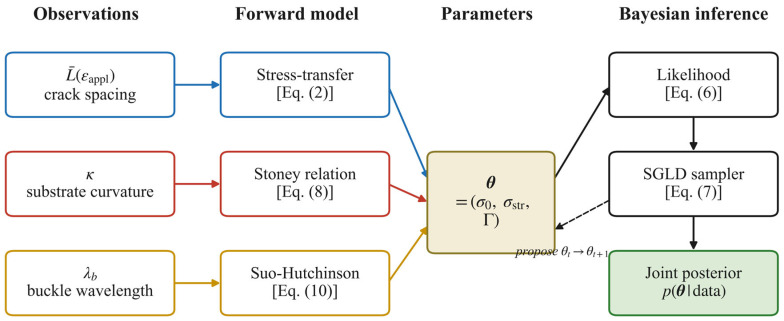
Bayesian physics-informed inversion framework. Three independent observation channels (the crack-spacing curve $\bar{L}(\varepsilon_{\mathrm{appl}})$, the substrate curvature κ, and the buckle half-wavelength $\lambda_{b}$) are connected to the shared parameter vector $\theta =(\sigma_{0},\;\sigma_{\mathrm{str}},\;\Gamma )$ through their respective forward models (stress-transfer, Stoney relation, and Suo–Hutchinson energy balance, Equations (2), (8), and (10)). The likelihood [Equation (6)] aggregates the residuals from all available channels, and stochastic gradient Langevin dynamics [Equation (7)] samples the joint posterior p(θ ∣ data). The dashed feedback line indicates the iterative update of $\theta_{t}$.

Figure 2 summarises the overall structure of the Bayesian inversion framework, in which each observation channel constrains the shared parameter vector $\theta =(\sigma_{0},\;\sigma_{\mathrm{str}},\;\Gamma )$ through its corresponding forward model.

### 3.1. Likelihood and Prior

For a film of given thickness, the experimental data consist of N pairs ${\left\{\left(\varepsilon_{i},\;{\bar{L}}_{i}\right)\right\}}_{i=1}^{N}$ of applied strain and measured the mean crack spacing. The measurement is modelled as the true value perturbed by multiplicative Gaussian noise with relative standard deviation $\sigma_{n}=0.08$, a value consistent with the scatter visible in Figure 10 of our earlier study [8].

(4) $$L_{i}^{obs}=L_{i}^{true}\left(\sigma_{0},\sigma_{str}\right)+N\left(0,\sigma_{n}^{2}L_{i}^{true\;2}\right)$$

Because Equation (3) defines ${\bar{L}}_{i}^{\mathrm{true}}$ only implicitly, evaluating Equation (4) directly would require an inner root-finding step at every Markov-chain iteration. We therefore work with a linearised form of the likelihood in which the data misfit is measured in stress rather than in length. Defining the dimensionless stress residual,

(5) $$r_{i}\left(\sigma_{0},\sigma_{str}\right)=\frac{\sigma_{x,max}^{f}\left(\sigma_{0},\varepsilon_{i},{\bar{L}}_{i}^{obs}\right)-\sigma_{str}}{\sigma_{str}}$$

the negative log-likelihood reduces to a sum of squared residuals,

(6) $$-logp\left(\left\{L_{i}\right\}|\sigma_{0},\sigma_{str}\right)=\frac{1}{2\sigma_{n}^{2}}\sum_{i=1}^{N}r_{i}^{2}+const$$

A broad, uniform prior is assigned to $\sigma_{0}$ over [−3, +3] GPa. Unless stated otherwise, $\sigma_{\mathrm{str}}$ is given a weakly informative Gaussian prior centred at 413 MPa with a standard deviation of 50 MPa. This prior is deliberately broader than the conventional fixed bulk-strength assumption, allowing the data to reveal departures arising from thin-film size effects or substrate constraint [1].

### 3.2. Posterior Sampling

The joint posterior is sampled using stochastic gradient Langevin dynamics [11],

(7) $$\theta_{t+1}=\theta_{t}-\eta \nabla_{\theta }U\left(\theta_{t}\right)+N\left(0,2\eta I\right),\;\;U=-logp\left(\theta |\left\{L_{i}\right\}\right)$$

with $\theta =(\sigma_{0},\;\sigma_{\mathrm{str}})$. Both components are updated in a normalised parameter space with preconditioned step sizes of $5\times 10^{-6}$. Each chain comprises a burn-in of 2000 steps followed by 4000 retained samples after thinning. Positivity of $\sigma_{\mathrm{str}}$ is enforced by reflection at zero.

### 3.3. Limiting Case: The Closed-Form Estimator

The closed-form estimator we proposed in [8] arises as a limiting case of the present Bayesian formulation (Appendix A). If $\sigma_{\mathrm{str}}$ is replaced by a Dirac prior centred at 413 MPa, and the likelihood is restricted to the single observation at the onset of cracking, Equation (6) collapses to the squared residual at that point. The maximum a posteriori estimate of $\sigma_{0}$ then coincides with the value that satisfies Equation (3) for $\sigma_{\mathrm{str}}=413$ MPa, which is precisely the closed-form solution we derived in our earlier work. The present framework, therefore, generalises our earlier estimator rather than substituting an unrelated surrogate for its underlying mechanics.

### 3.4. Numerical Implementation

The forward map is implemented in PyTorch (version 2.3.1) with automatic differentiation through the stress-transfer equations. The transcendental equation for $d_{2}$ is solved once per material system by bracketed bisection. Sampling for a single film thickness takes a few minutes on one CPU; the implementation accepts user-supplied experimental data through a CSV interface.

### 3.5. Network Architecture and Training

The forward map, defined by Equations (2), (8), and (10), is implemented in PyTorch with automatic differentiation and is physics-informed in two senses. First, every data channel enters the loss through its governing physical relation (the Yin–Li stress-transfer equation [Equation (2)], the Stoney relation [Equation (8)], or the Suo–Hutchinson energy balance [Equation (10)]), rather than through a generic regression surrogate. Second, a small neural network represents the in-plane film displacement field *u_f_*(*x*) along the mid-plane, and the one-dimensional strain–balance relation (*du_f_*)/(*dx*) = ((1 − *ν_f_*^2^)*σ_x_^f^*)/(*E_f_*) is imposed as a differential residual evaluated by automatic differentiation at collocation points; this residual acts as a soft physical regulariser that keeps the inversion on a mechanically admissible manifold.

Architecture: The displacement network is a fully connected multilayer perceptron with one input (the normalised mid-plane coordinate *x*/$\bar{L}$), three hidden layers of 24 units each, and two linear outputs (the in-plane and out-of-plane displacement components). All hidden layers use the hyperbolic-tangent (tanh) activation; the output layer is linear. The physical unknowns *σ*_0_, *σ*_str_, and *Γ* are carried as additional trainable scalar parameters, non-dimensionalised for conditioning (*σ*_0_ scaled by 10^9^ Pa, *Γ* expressed in J m^−2^). The strain–balance residual is sampled at 64 collocation points along the film mid-plane.

Loss and likelihood: The composite objective is *L* = (1)/(2*σ_n_*^2^)(*L_κ_* + $\bar{L}$ + *L*_buckle_) + 0.1,*L*_PDE_, where *L_κ_*, $\bar{L}$, and *L*_buckle_ are the relative-squared residuals of the curvature, crack-spacing, and buckle-wavelength channels, *L*_PDE_ is the strain–balance residual, and *σ_n_* is the relative noise measurement standard deviation. Scaling the data terms by 1/2*σ_n_*^2^ makes the negative Gaussian log-likelihood of the stated measurement model an objective, so that the optimiser locates the maximum a posteriori estimate and the sampler targets the correct posterior. We use *σ_n_* = 0.08 for crack spacing, which is consistent with the scatter in Figure 10 of [8], and *σ_n_* = 0.05 for the curvature and buckle channels.

Training and inference: Point estimates are obtained with Adam (using a learning rate of 5 × 10^−3^ and gradient-norm clipping at 5.0) for 2500–3000 iterations, followed by an L-BFGS refinement (using a strong-Wolfe line search, a history size of 30–50, and up to 100–200 iterations). Posterior uncertainty is then quantified by three complementary schemes that are cross-checked against one another as follows: (a) stochastic gradient Langevin dynamics [Equation (7)] on the physical parameters, with preconditioned per-parameter step sizes of 5 × 10^−6^ in the scaled space, a 2000–3000-step burn-in, 4000–8000 retained samples, and fivefold thinning, with positivity of *σ*_str_ and *Γ* enforced by reflection at zero; (b) deep ensembles of ten independently initialised members trained under independent noise measurement realisations; and (c) Monte Carlo dropout with a dropout probability of 0.05. The three schemes yield mutually consistent posteriors, indicating that the reported uncertainties are not artefacts of a single inference method.

Convergence and reproducibility: For each inversion, four independent chains were run using different initialisations; the Gelman–Rubin statistic is$\;\hat{R}\;$ ≤ 1.12, and the effective sample size is between 23 and 741 (Table S1), using the trace and autocorrelation diagnostics seen in Figures S3–S5. All runs use fixed random seeds; the forward model is evaluated in double precision and runs in minutes per thickness on a single CPU. The geometry coefficients are obtained once per material system by bracketed bisection to a tolerance of 10^−8^. The complete list of architectural and sampler hyperparameters is given in Table S5.

## 4. Application to Cr/Polyimide Fragmentation Data

### 4.1. Data and Inference Modes

The re-analysis uses the $\bar{L}(\varepsilon_{\mathrm{appl}})$ data digitised from Figure 10 of our earlier study [8] for 100, 200, and 400 nm Cr films on a 125 μm polyimide substrate. Six applied strain levels are retained for each thickness: 1.2%, 1.5%, 2.0%, 2.5%, 3.0%, and 3.4%. Strain levels below the cracking onset are excluded from the likelihood, since the forward model admits no physical crack-spacing solution in that regime.

Three inference modes are compared in order to isolate the influence of the fracture strength assumption and of the additional curvature channel. Mode A uses the crack-spacing curve alone, with the weakly informative prior on $\sigma_{\mathrm{str}}$ defined in Section 3.1. Mode B uses the same crack-spacing curve but fixes $\sigma_{\mathrm{str}}$ at 413 MPa, thereby reproducing the implicit assumption that underlies our previous closed-form analysis [8]. Mode C combines the crack-spacing curve with one synthetic Stoney-curvature observation, obtained by adding 5% Gaussian noise to the $\sigma_{0}$ value that we previously reported in [8]. The synthetic observation is intended to represent the information content of a single white-light interferometric scan and is used here in the absence of a directly measured curvature value.

### 4.2. Posterior Estimates and Identifiability

**Figure 3:**
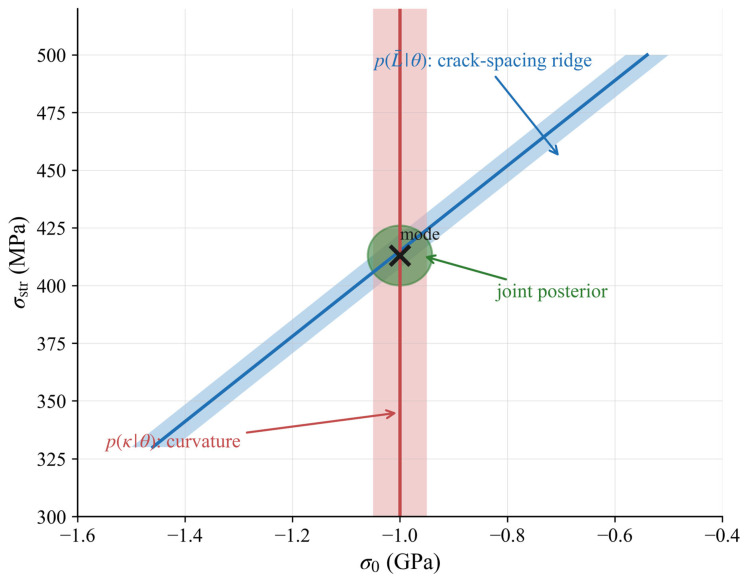
Identifiability geometry in the residual stress–fracture strength plane. Schematic likelihood ridges from individual observation channels and their joint posterior. The crack-spacing likelihood $p(\bar{L}\;|\;\theta )$ defines a narrow but elongated ridge along a diagonal manifold in the $\left(\sigma_{0},\sigma_{\mathrm{str}}\right)\;$ plane (blue band). The curvature likelihood p(κ ∣ θ), which, through the Stoney relation, depends on $\sigma_{0}$ but not on $\sigma_{\mathrm{str}}$ defines a nearly vertical band (red lines and shaded region). Their intersection yields the compact joint posterior (green) and identifies a unique mode (cross), thereby breaking the one-dimensional degeneracy of the crack-spacing channel.

Before presenting the numerical posterior estimates, it is useful to anticipate the geometric structure that they will exhibit. The identifiability problem underlying the present inversion can be understood schematically from Figure 3. The crack-spacing likelihood defines a narrow but elongated ridge along a diagonal manifold in the $\left(\sigma_{0},\;\sigma_{\mathrm{str}}\right)$ plane, reflecting the coupling expressed by Equation (3): for a given observed pair $\left(\bar{L},\;\varepsilon_{\mathrm{appl}}\right)$, an increase in $\sigma_{\mathrm{str}}$ can be largely compensated for by a corresponding shift in $\sigma_{0}$. The curvature likelihood, which through the Stoney relation depends on $\sigma_{0}$ alone, defines a nearly vertical band; its intersection with the crack-spacing ridge collapses the joint posterior to a compact, well-resolved region.

Table 1 summarises the posterior estimates obtained from the three inference modes defined in Section 4.1. Values are reported as posterior means ± one standard deviation unless stated otherwise.

**Figure 4:**
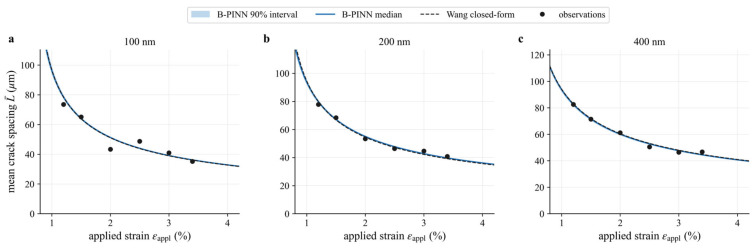
Posterior predictive checks for the Cr/polyimide fragmentation curves. Panels (**a**–**c**) correspond to 100, 200, and 400 nm Cr films, respectively. Black circular markers denote observations digitised from Figure 10 of [8]; the black dashed line is the closed-form prediction we derived in our earlier work; the blue solid line and shaded band are the Mode C posterior median and the 90% predictive interval.

Posterior predictive checks confirm that the inferred parameters reproduce the measured fragmentation curves (Figure 4). Across all three thicknesses, the Mode C predictive median follows the experimental trend and remains close to our earlier closed-form prediction. The narrow predictive band should not, however, be interpreted as evidence of unique parameter identifiability: many combinations of $\sigma_{0}$ and $\sigma_{\mathrm{str}}$ reproduce the crack-spacing data equally well, and the contraction observed in Mode C is entirely dependent on the curvature channel.

**Figure 5:**
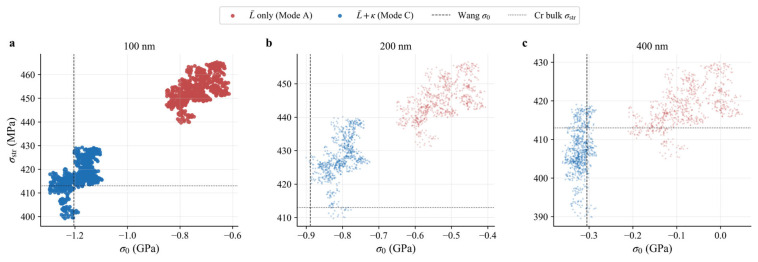
Joint posterior of $\left(\sigma_{0},\;\sigma_{\mathrm{str}}\right)$ under two inference modes. Red markers show the Mode A posterior, which uses the crack-spacing curve $\bar{L}(\varepsilon_{\mathrm{appl}})$ alone; blue markers show the Mode C posterior, which additionally incorporates one Stoney-curvature observation. Vertical dashed lines mark the residual stresses we previously reported in [8]; the horizontal dotted line marks the bulk fracture strength of Cr (413 MPa). Panels (**a**–**c**) correspond to 100, 200, and 400 nm Cr films, respectively.

The numerical posterior samples shown in Figure 5 confirm the geometric picture anticipated in Figure 3. When only the crack-spacing curve is used (Mode A), the posterior is elongated along a diagonal manifold, which is the uncertainty structure that is hidden when $\sigma_{\mathrm{str}}$ is prescribed a priori. Adding the curvature observation (Mode C) contracts the posterior toward the residual stress that we previously reported in [8] and toward the bulk fracture strength of Cr. This is because the Stoney relation depends on $\sigma_{0}$ but is insensitive to $\sigma_{\mathrm{str}}$.

**Figure 6:**
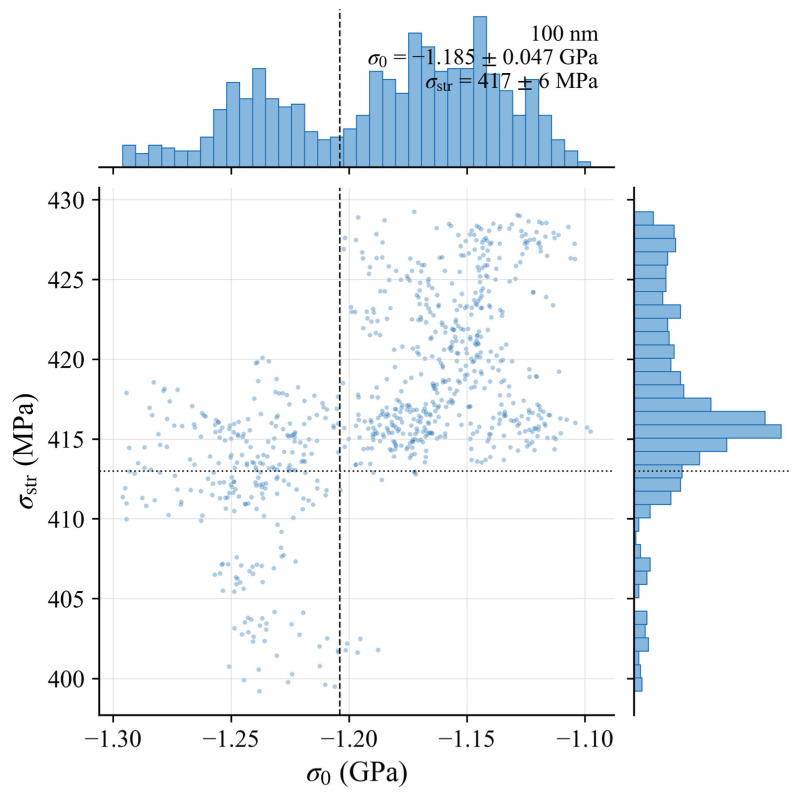
Mode C joint posterior for the 100 nm Cr/polyimide film. The central scatter plot shows the joint posterior of $\sigma_{0}$ and $\sigma_{\mathrm{str}}$. The histograms above and to the right show the corresponding marginal distributions. The vertical dashed line marks the residual stress that we previously reported in [8], and the horizontal dotted line marks the Cr bulk fracture strength of 413 MPa. Posterior means are indicated in the upper-right annotation. The corresponding corner plots for the 200 and 400 nm films are provided in the Supplementary Material.

Per-thickness corner plots of the Mode C posterior are provided in Figure 6. The marginal distributions of $\sigma_{0}$ and $\sigma_{\mathrm{str}}$ are unimodal and concentrated near the bulk values, and the residual diagonal correlation is markedly reduced once the curvature observation is included.

Taken together, the numerical estimates in Table 1 and the posterior geometry in Figure 5 support three conclusions. First, the $\bar{L}(\varepsilon_{\mathrm{appl}})$ data alone are insufficient to identify a unique pair $\left(\sigma_{0},\;\sigma_{\mathrm{str}}\right)$: under the weakly informative fracture strength prior, Mode A displaces $\sigma_{0}$ from our previously reported value by between 39% and 74%, with a compensating increase in $\sigma_{\mathrm{str}}$. Second, fixing $\sigma_{\mathrm{str}}$ in Mode B substantially recovers our earlier estimate, which confirms that the present Bayesian implementation is mechanically consistent with our earlier closed-form estimator. Third, the addition of curvature in Mode C breaks the degeneracy: the resulting residual stresses agree with our earlier values to within 1.6–9.4%, while the inferred fracture strengths lie within 1.5–4% of the Cr bulk value.

The methodological implication is significant and applies, in particular, to our own earlier work. The value of $\sigma_{0}$ that we previously reported in [8] is not, in itself, a quantity extracted purely from fragmentation data; it is the joint outcome of fragmentation data combined with an a priori prescription of the fracture strength. The Bayesian formulation developed here makes this dependence explicit and identifies the additional observation needed to recover both quantities from data alone.

The robustness of these estimates to the modelling choices was examined systematically. Varying the prior standard deviation on *σ*_str_ over 25–100 MPa changes the posterior mean of *σ*_0_ negligibly: the change is smaller than the posterior standard deviation, and the 90% credible interval always contains the previously reported value. The *σ*_str_ posterior width, by contrast, scales with the prior width, the expected signature of the identifiability structure described above, in which the curvature channel constrains *σ*_0_ independently of the prior while *σ*_str_ remains partially prior-informed because the crack-spacing curve alone does not fully determine it. Varying the assumed relative crack-spacing noise over a four-fold range (*σ_n_* = 0.04–0.16) likewise leaves the posterior means stable and broadens the credible intervals approximately linearly, as expected for a Gaussian likelihood without strong prior tension. All inversions pass standard convergence checks, with a Gelman–Rubin statistic of R ≤ 1.12 across four independent chains. Full results are given in Supplementary Sections S3 and S4 and Tables S1–S3.

## 5. Multi-Channel Extension and Identifiability of the Interface Adhesion Energy

The Bayesian framework presented in Section 3 admits additional independent observation channels. Substrate curvature provides, via the Stoney relation,

(8) $$\kappa =\frac{6\sigma_{0}h_{f}}{E_{s}^{\prime }h_{s}^{2}},\;\;E_{s}^{\prime }=\frac{E_{s}}{1-\nu_{s}^{2}}$$

applies a direct linear constraint on $\sigma_{0}$ that is insensitive to $\sigma_{\mathrm{str}}$. If, in addition, the film develops steady-state buckles [14,15,16], the buckle half-wavelength $\lambda_{b}$ is related to the critical buckling stress by

(9) $$\sigma_{c}=\frac{\pi^{2}}{12}\frac{E_{f}}{1-\nu_{f}^{2}}{\left(\frac{h_{f}}{\lambda_{b}}\right)}^{2}$$

and the steady-state energy-release condition of Suo and Hutchinson [14,15] gives,

(10) $$G_{ss}=\frac{(1-\nu_{f}^{2})h_{f}}{2E_{f}}\left(|\sigma_{0}|-\sigma_{c}\right)\left(|\sigma_{0}|+3\sigma_{c}\right)=\Gamma$$

where $|\sigma_{0}|$ denotes the magnitude of the compressive residual stress.

The three observation channels, therefore, separate the unknowns by physical content: the curvature and crack-spacing data jointly identify $\sigma_{0}$ and $\sigma_{\mathrm{str}}$, while the buckle wavelength supplies the additional information needed to infer the interface adhesion energy Γ, provided that a physical steady-state buckle solution exists.

The feasibility region for this third channel can be made explicit. Introducing the ratio $r=\sigma_{c}/|\sigma_{0}|$ and maximising the right-hand side of Equation (10) with respect to r on the admissible interval 0<r<1  yields the maximum at r=1/3:

(11) $$\Gamma_{max}\left(h_{f},\sigma_{0};E_{f},\nu_{f}\right)=\frac{4}{3}\frac{\left(1-\nu_{f}^{2}\right)h_{f}\sigma_{0}^{2}}{2E_{f}}$$

**Figure 7:**
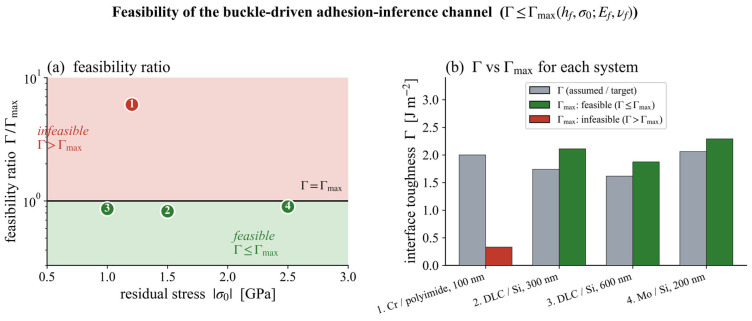
Feasibility of the buckle-wavelength channel for inferring the interface adhesion energy. A steady-state buckle exists only when *Γ* ≤ *Γ*_max_ = (4)/(3)((1 − *ν_f_*^2^)*h_f_σ*_0_^2^)/(2*E_f_*) (Equation (11)); because *Γ*_max_ depends on the film thickness and modulus, feasibility is shown per system rather than on a single curve. (**a**) Dimensionless feasibility ratio *Γ*/*Γ*_max_ versus residual stress magnitude |*σ*_0_| (logarithmic scale); the horizontal line marks *Γ* = *Γ*_max_, and the feasible region (*Γ* ≤ *Γ*_max_) lies below it. (**b**) The assumed or target adhesion energy *Γ* compared with each system’s own *Γ*_max_ from Equation (11). The four systems are as follows: ① Cr/polyimide (infeasible, *Γ*/*Γ*_max_ ≈ 6), and ②–④ are the synthetic DLC/Si and Mo/Si systems (feasible, *Γ*/*Γ*_max_ ≈ 0.8–0.9).

A real steady-state buckle wavelength, therefore, exists only when $\Gamma \leq \Gamma_{max}$. The resulting feasibility region is shown in Figure 7, together with the projected positions of the four systems considered in this work. For the 100 nm Cr/polyimide specimen with $\sigma_{0}\approx -1.2$ GPa, Equation (11) gives $\Gamma_{max}\approx 0.33\;{J\;m}^{-2}$, which lies well below the 1–10 ${J\;m}^{-2}$ range that is typical of metal/polymer interfaces. The Cr/polyimide specimens analysed in our earlier work [8], therefore, do not satisfy the feasibility condition for buckle-based adhesion recovery, a fact made visually explicit by the position of marker ① in Figure 7. Accordingly, the buckle-wavelength channel is introduced here as a methodological extension demonstrating the identifiability of Γ in principle rather than as a basis for inferring the interface adhesion energy of the specimens previously analysed in [8]. As such, an explicit validation of the three-channel inversion is provided on independent synthetic systems in Section 6.

## 6. Synthetic Proof-of-Concept of the Three-Channel Extension

To validate the three-channel inversion in regimes where the feasibility condition of Equation (11) is satisfied, we generate synthetic curvature, crack-spacing, and buckle-wavelength observations for two representative thin-film systems: diamond-like-carbon (DLC) films on Si [17], and a Mo film on Si [18]. Two DLC/Si specimens are considered, with film thicknesses of 300 and 600 nm, target residual stresses of −1.500  and −1.000 GPa, and target interface adhesion energies of 1.740 and 1.617 ${J\;m}^{-2}$, respectively. The Mo/Si specimen has a film thickness of 200 nm, a target residual stress of −2.500 GPa, and a target adhesion energy of 2.061 ${J\;m}^{-2}$. Each observation channel is perturbed by independent multiplicative Gaussian noise with a relative standard deviation of 5% (Table 2).

**Figure 8:**
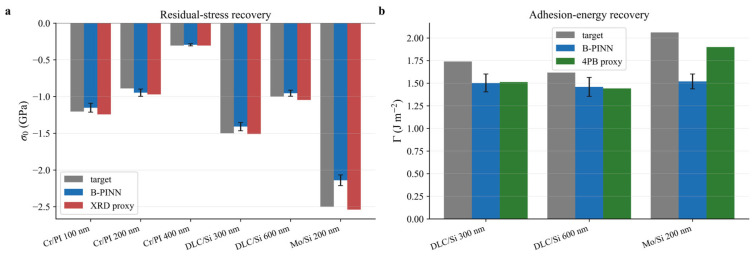
Cross-method comparison of recovered and target values for the three-channel synthetic validation. (**a**) Residual stress $\sigma_{0}\;$for the Cr/polyimide, DLC/Si, and Mo/Si specimens, comparing the target value (grey), the B-PINN posterior estimate (blue), and an XRD proxy (red). (**b**) Interface adhesion energy Γ for the DLC/Si and Mo/Si specimens in which buckling is feasible, comparing the target value (grey), the B-PINN posterior estimate (blue), and a four-point-bending (4PB) proxy reconstructed from [19,20] (green). Error bars on the B-PINN bars denote 90% credible intervals obtained from the joint posterior of the three-channel inversion.

A cross-method summary of the validation is presented in Figure 8, in which the B-PINN posterior estimates are compared with conventional measurement proxies (an XRD-style proxy for $\sigma_{0}$ and a four-point-bending (4PB) proxy for Γ). Figure 8a additionally includes the Cr/polyimide residual stresses obtained in Section 4 to facilitate cross-comparison; Figure 8b is restricted to the three synthetic systems for which the buckle channel is feasible. The residual stress is recovered most accurately since both the curvature and the crack-spacing channels independently constrain $\sigma_{0}$. The adhesion energy is recovered with a small systematic low bias, which is most pronounced for the Mo/Si specimen (approximately 26%, against 14% and 10% for the DLC/Si–300 and DLC/Si–600 specimens). This bias arises from the negative coupling between $\sigma_{0}$ and Γ in the Suo–Hutchinson relation (Equation (10)) and therefore reflects a structural feature of the inverse problem rather than a numerical artefact of the sampler. In practice, a measurable discrepancy between the B-PINN estimate of Γ and an independently obtained value (for example, from four-point-bending experiments [19,20]) would indicate one of three contributions: observation noise, branch ambiguity in solving Equation (10) for $\sigma_{c}$, or a mismatch between the ideal steady-state buckle model and the actual delamination morphology of the specimen.

The systematic negative bias in the recovered adhesion energy (approximately 26% for Mo/Si, and 14% and 10% for the DLC/Si-300 and DLC/Si-600 systems) originates in the structure of the inverse problem rather than in the sampler or the noise model. Introducing the ratio *r* = *σ_c_*/|*σ*_0_| of the critical buckling stress to the residual stress magnitude, the Suo–Hutchinson balance [Equation (10)] can be written as *G*_ss_ = ((1 − *ν_f_*^2^)*h_f_σ*_0_^2^)/(2*E_f_*)(1 − *r*)(1 + 3*r*) = *Γ*. For a fully developed, experimentally observable buckle, the critical stress is small relative to the residual stress (*r* → 0), the geometric factor (1 − *r*)(1 + 3*r*) tends to unity, and *Γ* → ((1 − *ν_f_*^2^)*h_f_σ*_0_^2^)/(2*E_f_*); that is, *Γ* ∝ *σ*_0_^2^. The logarithmic sensitivity is therefore *∂Γ*/*∂*|*σ*_0_| ≈ 2, so a fractional error in the recovered residual stress propagates into a fractional error of roughly twice the magnitude (and of the same sign) in the recovered adhesion energy.

This prediction is quantitative. Squaring the ratio of the recovered to the true residual stress magnitude gives expected adhesion biases of −11.8%, −8.8%, and −26.7% for DLC/Si-300, DLC/Si-600, and Mo/Si, which are in close agreement with the observed −13.7%, −9.8%, and −26.3%. Mo/Si is the most strongly affected simply because it has the largest residual stress magnitude (2.5 GPa); therefore, the same fractional residual stress uncertainty produces the largest squared error. The residual discrepancy between the (ratio)^2^ prediction and the observed bias (for example, −26.7% versus −26.3% for Mo/Si) is a second-order contribution from the finite critical stress factor (1 − *r*)(1 + 3*r*) and from the selection of the buckle branch, which are both small compared with the leading *σ*_0_^2^ term.

The noise measurement is not the cause of the systematic sign. The synthetic noise is zero-mean multiplicative Gaussian and therefore contributes scatter (the width of the posterior credible interval) but not a one-sided offset; reducing the noise narrows the credible interval, while the posterior mean of *Γ* continues to track the square of the recovered *σ*_0_. Because the bias is inherited from *σ*_0_ and amplified by the factor of two, it is reduced by any measurement that tightens *σ*_0_, or by correcting for the known mapping as follows: (i) adding the independent, low-uncertainty curvature channel exploited in Section 4 (halving the *σ*_0_ error halves the *Γ* error); (ii) reducing the buckle-wavelength noise measurement; and (iii) applying an analytic first-order correction by using the closed-form derivative *∂Γ*/*∂σ*_0_ = ((1 − *ν_f_*^2^)*h_f_σ*_0_)/(*E_f_*).

## 7. Discussion

Before discussing the implications of our results, we state the assumptions of the forward model and the level of evidence that support each claim. The inversion inherits the mechanical assumptions of the Wang21 stress-transfer model [8] as follows: (i) two-dimensional plane strain; (ii) linear isotropic elasticity for film and substrate; (iii) a perfectly bonded interface away from cracks and buckles; (iv) the Yin–Li analytic stress field for the maximum tensile film stress [Equation (2)]; (v) a brittle, strength-controlled fragmentation criterion [Equation (3)]; (vi) the Stoney relation for the curvature channel [Equation (8)], valid in the thin-film limit *h_f_* ≪ *h_s_*; and (vii) the Suo–Hutchinson steady-state energy balance for the buckle channel [Equation (10)]. Departures from these assumptions (finite specimen width, substrate plasticity at large strain, anisotropic columnar microstructure, or non-steady-state buckle morphology) define the domain of validity of the present results and motivate the extensions discussed below.

The three observation channels are not on an equal experimental footing. The residual stress (*σ*_0_) and fracture strength (*σ*_str_) identification in Section 3 and Section 4 are the result of a re-analysis of real fragmentation data: the crack-spacing curves $\bar{L}$(*ε*_appl_) for the 100, 200, and 400 nm Cr films are digitised from measured fragmentation experiments (Fig 10 of [8]), and the curvature channel corresponds to a single, standard, non-destructive wafer-curvature measurement that is obtainable on the same specimen class. Across the three thicknesses, this part of the framework recovers the previously reported residual stresses to within 1.6–9.4%, while simultaneously inferring a fracture strength within 1.5–4% of the bulk Cr value of 413 MPa without prescribing it a priori. The interface adhesion energy (*Γ*) identification in Section 5 and Section 6, by contrast, is supported by synthetic data only: as the feasibility analysis of Section 5 shows, the available Cr/polyimide specimens cannot form steady-state buckles on which that channel relies. We therefore present the adhesion energy channel as a methodological capability whose identifiability is established in principle and quantified on synthetic systems, and not as an experimentally validated measurement (Table 3).

The practical reliability of the adhesion energy channel is, at present, established only in silico, and three factors will govern its reliability in a real experiment. First, because the Suo–Hutchinson balance [Equation (10)] couples *Γ* to *σ*_0_ quadratically (Section 6), the relative uncertainty in *Γ* is approximately twice that in *σ*_0_; an independent, low-uncertainty *σ*_0_ measurement, most naturally the wafer curvature channel already used in this work, is therefore a prerequisite for an accurate *Γ*. Second, Equation (10) is quadratic in the critical buckling stress *σ_c_* and admits two roots; the physically developed buckle branch (*σ_c_* ≪ |*σ*_0_|) must be selected from the observed buckle morphology, and a wrong branch choice biases *Γ*. Third, the model assumes an ideal, straight-sided steady-state buckle, whereas real delaminations are frequently telephone-cord shaped; the wavelength entering Equation (9) must therefore be extracted in a manner that is consistent with the idealisation or the model generalised to the observed morphology.

The results clarify what has been experimentally validated and what remains a methodological proposal. The Cr/polyimide component of this study is a re-analysis of previously published fragmentation curves [8] using real data. It validates the Bayesian recovery of $\sigma_{0}$ within the original stress-transfer mechanics of our earlier work and demonstrates how uncertainty in $\sigma_{\mathrm{str}}$ shapes the inference. The curvature channel is evaluated as a minimal additional observation that can be acquired non-destructively on the same class of specimens. The adhesion energy extension, by contrast, is not validated on the Cr/polyimide specimens themselves since the feasibility condition derived in Equation (11) shows that steady-state buckling is unavailable under the published film thicknesses and stress range.

The present approach has different strengths and weaknesses relative to existing characterisation techniques. Compared with X-ray diffraction [2], it does not require crystalline films and is considerably less expensive to deploy, but it does remain model-dependent. Compared with wafer curvature alone [4], it additionally provides access to the film fracture strength through fragmentation and, in geometries where buckling is feasible, to the interface adhesion energy. Compared with four-point bending [19,20], it is non-destructive, although the adhesion estimate is conditional on correct identification of the Suo–Hutchinson buckle branch [14,15]. The framework is therefore best viewed as a transparent inverse formulation that integrates multiple low-cost observations rather than as a universal replacement for specialised stress or adhesion measurements.

Several limitations should guide future applications. The forward model assumes plane strain and linear elasticity; finite-width specimens, nonlinear substrate deformation at applied strains above a few per cent, and anisotropic columnar microstructures may require a differentiable finite element forward model. The synthetic adhesion energy examples further assume known elastic constants and globally interpretable buckle wavelengths; in actual experiments, uncertainty in the elastic moduli, local variations in the delamination morphology, and noise in the image-based extraction of $\lambda_{b}$ should be explicitly propagated into the posterior.

Three natural extensions of the framework are envisaged. First, hierarchical Bayesian pooling could share fracture strength information across films of different thickness produced from the same material system. Second, differentiable finite element solvers could replace the analytical stress-transfer equations to accommodate arbitrary specimen geometries. Third, in situ digital image correlation could add a spatial strain-field channel that further reduces parameter degeneracy without recourse to destructive testing.

### 7.1. Toward an Experimental Validation of the Three-Channel Model

A steady-state buckle exists only when the stored elastic strain energy is large relative to the interface toughness, i.e., when *Γ* ≤ *Γ* = (4)/(3)((1 − *ν_f_*^2^)*h_f_σ*_0_^2^)/(2*E_f_*) [Equation (11)]. A decisive real-data test of the three-channel model, therefore, requires a specimen that combines a stiff, strongly compressed, brittle film (to make *Γ* large) with a comparatively weak interface (to make *Γ* small). Suitable systems include sputter-deposited diamond-like carbon, Mo, TiN, CrN, or W films of 100–500 nm thicknesses, with a compressive residual stress |*σ*_0_| ≈ 1.5–3 GPa and a modulus *E_f_* ≈ 200–350 GPa, deposited, for example, on oxidised Si or glass without an adhesion-promoting interlayer so that the interface toughness lies in the accessible range of *Γ* ≈ 1–5 J m^−2^.

A worked example illustrates the margin. For a 400 nm diamond-like-carbon film (*E_f_* ≈ 200 GPa, *ν_f_* ≈ 0.25) at a compressive residual stress of |*σ*_0_| ≈ 2 GPa, Equation (11) gives *Γ* ≈ 5.0 J m^−2^, which is comfortably above a typical DLC/Si interface toughness of 1–5 J m^−2^; therefore, a developed buckle is expected, and the channel is informative. By contrast, the 100 nm Cr/polyimide specimen has *Γ* ≈ 0.33 J m^−2^, which is far below the 1–10 J m^−2^ of a metal/polymer interface, which is precisely why it is infeasible (marker ① in Figure 7).

On such a specimen, these three observables can all be acquired non-destructively on the same sample: the wafer curvature *κ*, by optical profilometry or a curvature scanner, yields *σ*_0_ through the Stoney relation; the crack-spacing curve $\bar{L}$(*ε*_appl_), recorded by in situ optical or scanning electron microscopy during a tensile/fragmentation test, yields *σ*_str_; and the steady-state buckle half-wavelength *λ_b_*, measured by white-light interferometry, atomic-force microscopy, or scanning electron microscopy, yields *Γ*. An independent four-point-bending or superlayer-indentation measurement of *Γ* would provide the ground-truth reference for cross-validation. This is precisely the configuration in which the synthetic DLC/Si and Mo/Si demonstrations of Section 6 would become a real-data validation, and it constitutes the natural next step of this work.

## 8. Conclusions

This study reformulates the closed-form residual stress estimator for brittle-film fragmentation that we previously developed in [8] as a Bayesian physics-informed inverse problem. The formulation preserves the stress-transfer mechanics of that earlier work and recovers the original estimator as a limiting case while exposing the uncertainty that is concealed when a single observation is combined with an a priori fixed fracture strength.

The re-analysis of the Cr/polyimide fragmentation curves of [8] shows that crack-spacing data alone constrain an elongated posterior manifold in the $\left(\sigma_{0},\;\sigma_{\mathrm{str}}\right)$ plane. A single Stoney-curvature observation supplies the missing orthogonal constraint, recovers our previously reported residual stresses to within 1.6–9.4%, and yields estimates of $\sigma_{\mathrm{str}}$ close to the Cr bulk value of 413 MPa without prescribing it in advance.

The feasibility condition derived in Equation (11) specifies when a buckle-wavelength observation can be added as a third channel for inferring the interface adhesion energy. This condition is not met by the Cr/polyimide specimens of [8] but is satisfied by the synthetic DLC/Si and Mo/Si systems considered in Section 6; on these systems, the three-channel inversion recovers both the residual stress and the interface adhesion energy, providing a synthetic proof-of-concept whose experimental validation we identify as the next step (Section 7.1). Overall, the proposed workflow converts a single-point estimator into a multi-observation, uncertainty-aware inverse framework for thin-film mechanics.

## Figures and Tables

**Table 1:** Bayesian re-analysis of the Cr/polyimide fragmentation curves previously reported in our earlier work [8]. The column $\sigma_{0}^{\mathrm{prev}}$ denotes the residual stress we previously reported in [8].

		Mode A ($\bar{L}$ Only)		Mode B (Fixed $\sigma_{\mathrm{str}}$)	Mode C ($\bar{L}+\kappa$)		
$h_{f}$ (nm)	$\sigma_{0}^{prev}$ (GPa)	$\sigma_{0}$ (GPa)	$\sigma_{\mathrm{str}}$ (MPa)	$\sigma_{0}$ (GPa)	$\sigma_{0}$ (GPa)	$\sigma_{\mathrm{str}}$ (MPa)	$\left|∆\sigma_{0}\right|/\sigma_{0}^{prev}$
100	−1.204	−0.735 ± 0.059	454 ± 5	−1.111 ± 0.043	−1.185 ± 0.047	417 ± 6	1.6%
200	−0.889	−0.529 ± 0.060	445 ± 5	−0.863 ± 0.043	−0.805 ± 0.035	428 ± 6	9.4%
400	−0.305	−0.078 ± 0.062	419 ± 5	−0.204 ± 0.044	−0.320 ± 0.015	407 ± 6	5.0%

**Table 2:** Posterior estimates from the three-channel Bayesian inversion on synthetic DLC/Si and Mo/Si data. Values are reported as posterior means ± one standard deviation; 90% credible intervals are obtained from the joint posterior.

		$\sigma_{0}$ (GPa)			Γ (J m^−2^)		
System	$h_{f}$ (nm)	Target	Posterior	90% CI	Target	Posterior	90% CI
DLC/Si-300	300	−1.500	−1.409 ± 0.057	[−1.495, −1.313]	1.740	1.502 ± 0.099	[1.340, 1.657]
DLC/Si-600	600	−1.000	−0.955 ± 0.041	[−1.021, −0.884]	1.617	1.459 ± 0.105	[1.276, 1.616]
Mo/Si-200	200	−2.500	−2.140 ± 0.072	[−2.252, −2.020]	2.061	1.519 ± 0.082	[1.386, 1.654]

**Table 3:** Level of evidence supporting each inferred quantity.

Quantity	Observation Channels	Data Type	Validation Outcome
Residual stress σ_0_	crack spacing + curvature	real (digitised from [8])	recovered to 1.6–9.4% of [8]
Fracture strength σ_str	crack spacing + curvature	real	within 1.5–4% of bulk, not prescribed
Adhesion energy Γ	curvature + crack spacing + buckle wavelength	synthetic (DLC/Si, Mo/Si)	identifiability shown; validation pending (Section 7.1)

## Data Availability

The original contributions presented in this study are included in the article/Supplementary Material. Further inquiries can be directed to the corresponding authors.

## Supplementary Materials

The following supporting information can be downloaded at: https://www.mdpi.com/article/10.3390/ma19132824/s1.

## Appendix A. Reduction to the Closed-Form Estimator

With $\sigma_{\mathrm{str}}$ fixed at 413 MPa, and only the onset observation $\left({\bar{L}}_{*},\;\varepsilon_{*}\right)\;$ retained, the posterior reduces to

(A1) $$p\left(\sigma_{0}\;|\;{\bar{L}}_{*},\;\varepsilon_{*}\right)\propto \;exp\left[-\frac{r_{*}^{\;2}\left(\sigma_{0}\right)}{2\sigma_{n}^{\;2}}\right],r_{*}\left(\sigma_{0}\right)=\frac{\sigma_{x,\max}^{f}\left(\sigma_{0},\;\varepsilon_{*},\;{\bar{L}}_{*}\right)-413\;\mathrm{MPa}}{413\;\mathrm{MPa}}$$

The maximum of this posterior occurs at $r_{*}(\sigma_{0})=0$; that is, at the value of $\sigma_{0}$ for which

(A2) $$\sigma_{x,max}^{f}\left(\sigma_{0},\;\varepsilon_{*},\;{\bar{L}}_{*}\right)=\;413\;\mathrm{MPa}.$$

Equation (A2) is precisely Equation (3) evaluated at the onset observation with $\sigma_{\mathrm{str}}\;\;$ fixed at the bulk value, and it coincides with the closed-form solution we derived in our previous work [8]. The earlier estimator is therefore the maximum a posteriori solution of the present Bayesian model under a single-point likelihood and a Dirac prior on the fracture strength.
