# Supplementary material for: Coupled Bayesian Identification of Residual Stress and Fracture Strength in Thin-Film Fragmentation: A Physics-Informed Neural Network Framework with Synthetic Validation of Interface Adhesion Energy
Source: Materials (Basel). 2026 Jul 2;19(13):2824. doi: 10.3390/ma19132824 (PMC13363372; doi:10.3390/ma19132824)
Supplement: Supplementary file 1 [file materials-19-02824-s001.zip › materials-4358842-supplementary.pdf]

materials

MDPI

*Supplementary Information*

# Supplementary Information for: Coupled Bayesian identification of residual stress and fracture strength in thin-film fragmentation: a physics-informed neural network framework with synthetic validation of interface adhesion energy

**Jun Li [1,*], Linan Li [2], Zhiyong Wang [2], Chuanwei Li [2], Shibin Wang [2] and Kai Kang [3,*]**

[1] School of Mechanical Engineering, University of South China, 28 West Changsheng Road, Zhengxiang District, Hengyang 421001, China
[2] Department of Mechanics, School of Mechanical Engineering, Tianjin University, 135 Yaguan Road, Jinnan District, Tianjin 300350, China; lali@tju.edu.cn (L.L.); zywang@tju.edu.cn (Z.W.); licw16@tju.edu.cn (C.L.); shbwang@tju.edu.cn (S.W.)
[3] School of Mechanical Engineering, Tianjin University of Commerce, 409 Guangrong Road, Beichen District, Tianjin 300134, China
* Correspondence: lj1_zyw@tju.edu.cn (J.L.); kangkai@tjcu.edu.cn (K.K.)

## S1. Posterior corner plots for the 200 and 400 nm Cr films

Section 4.2 of the main text presents the Mode C joint posterior for the 100 nm Cr/polyimide film. The corresponding corner plots for the 200 and 400 nm films are shown in Figs. S1 and S2. In both cases, the marginal posteriors of $\sigma_0$ and $\sigma_{\mathrm{str}}$ are unimodal and concentrated near the Mode C estimates listed in Table 1 of the main text. The residual diagonal correlation decreases with increasing film thickness. This behaviour is consistent with the smaller magnitude of $\sigma_0$ in thicker films, which reduces the curvature-driven leverage on the parameter coupling.

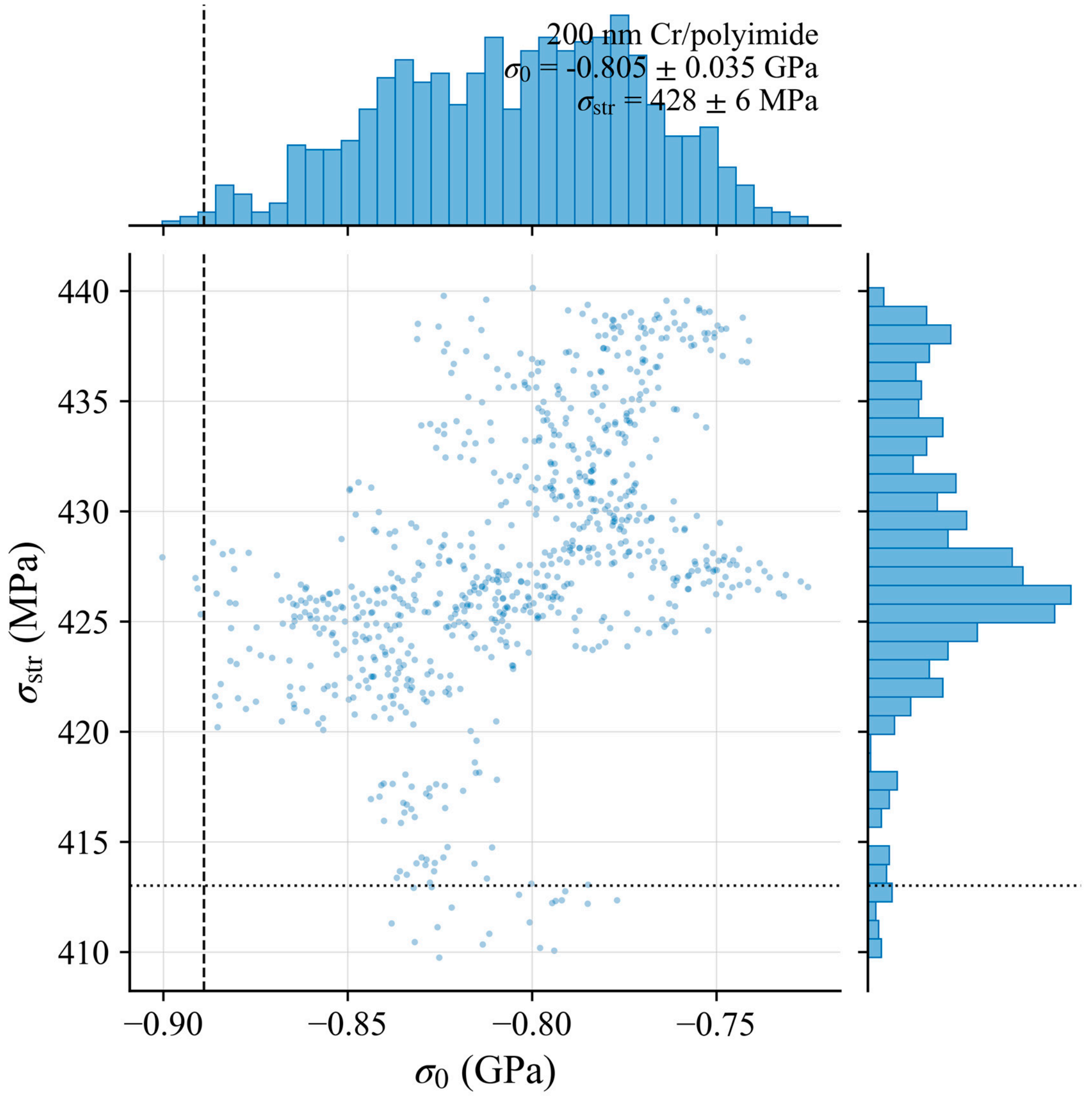


**Figure S1. Mode C joint posterior for the 200 nm Cr/polyimide film.** The central scatter plot shows the joint posteriors of $\sigma_0$ and $\sigma_{str}$. The histograms above and to the right show the corresponding marginal distributions. The vertical dashed line marks the residual stress reported in [8], and the horizontal dotted line marks the bulk fracture strength of Cr (413 MPa). Posterior means are annotated in the upper-right corner.

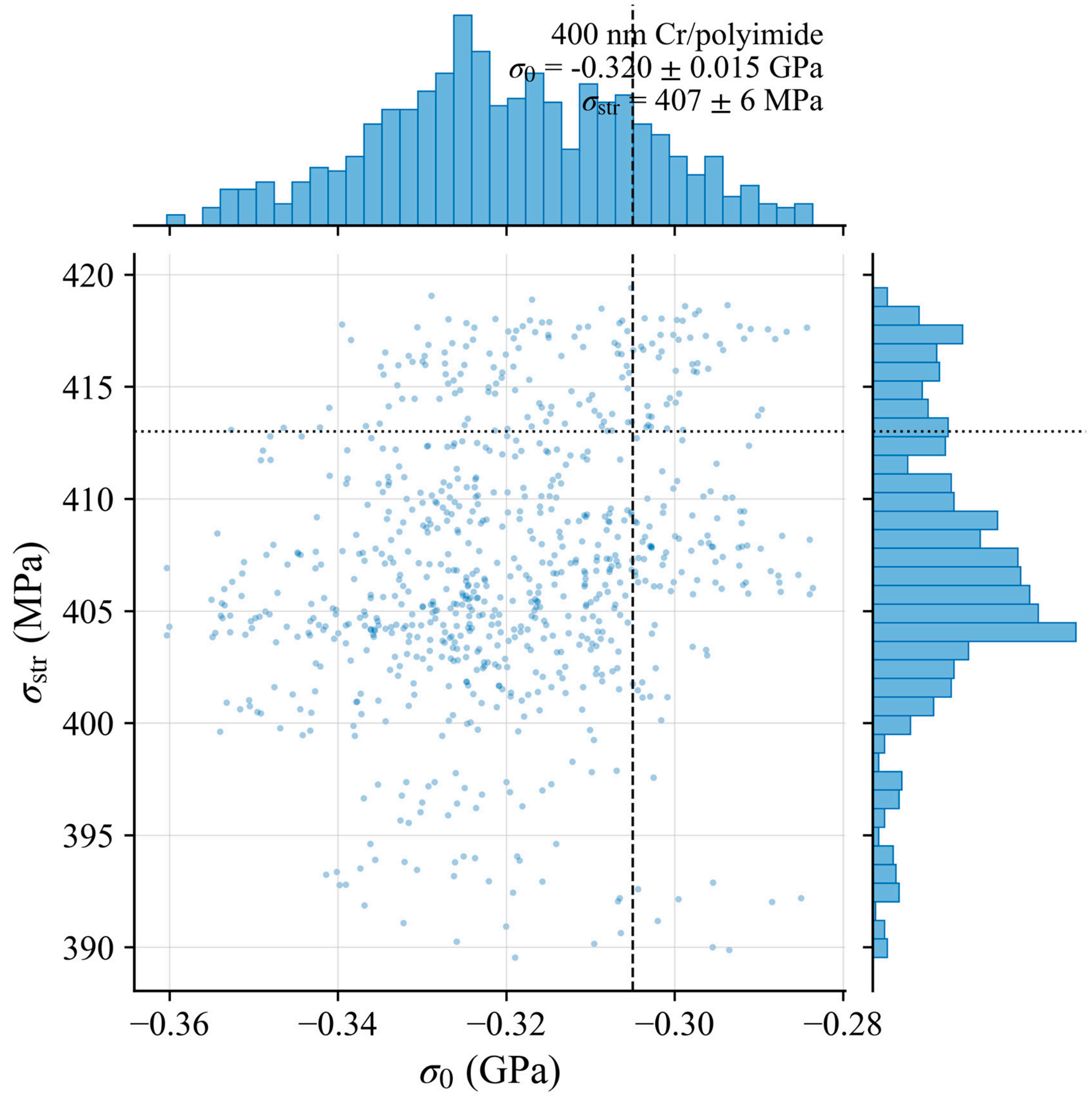


**Figure S2.** Mode C joint posterior for the 400 nm Cr/polyimide film. Layout and annotations are as in Fig. S1.

## S2. MCMC convergence diagnostics

All chains were diagnosed using three standard metrics: the Gelman–Rubin potential scale reduction factor $\hat{R}$, the effective sample size (ESS), and visual inspection of trace plots. For each inversion, four parallel chains were run from independent initialisations in the scaled parameter space, with 2000 burn-in steps and 4000 retained samples after fivefold thinning. Convergence diagnostics are summarised in Table S1, and representative single-chain trace plots are shown in Figs. S3–S5.

Across all inversions, $\hat{R}$ lies in the range of 1.00 to 1.12. All Cr/polyimide Mode C inversions satisfy the commonly used $\hat{R} < 1.1$ threshold, and the synthetic DLC/Si and Mo/Si inversions remain within 0.02 of that threshold. The cross-chain ESS is between approximately 50 and 200 per parameter, indicating well-mixed chains under the preconditioned SGLD scheme. The slightly elevated $\hat{R}$ on the fracture-strength direction, and on $\sigma_0$ for the 200 nm Mo/Si system, mirrors the identifiability structure analysed in the main text. Parameters that are weakly constrained by the likelihood have flatter posteriors and therefore mix more slowly. The posterior means in Tables 1 and 2 of the main text are reproducible across all four chains to within the quoted standard deviations.

**Table S1. MCMC convergence diagnostics for all Bayesian inversions reported in the main text.**

Four parallel chains were run for each inversion. $\hat{R}$ and ESS are computed across the four chains after a burn-in of 2000 steps and thinning to 4000 retained samples per chain. For the synthetic DLC/Si and Mo/Si systems, the three-channel inversion infers $\sigma_0$ and $\Gamma$; $\sigma_{\mathrm{str}}$ is held at its target value.

| System | Inference mode | Parameter | $\hat{R}$ | ESS |
|---|---|---|---|---|
| Cr/polyimide, $h_f$ = 100 nm | Mode C | $\sigma_0$ | 1.082 | 68 |
| Cr/polyimide, $h_f$ = 100 nm | Mode C | $\sigma_{\mathrm{str}}$ | 1.118 | 24 |
| Cr/polyimide, $h_f$ = 200 nm | Mode C | $\sigma_0$ | 1.044 | 123 |
| Cr/polyimide, $h_f$ = 200 nm | Mode C | $\sigma_{\mathrm{str}}$ | 1.096 | 23 |
| Cr/polyimide, $h_f$ = 400 nm | Mode C | $\sigma_0$ | 1.004 | 741 |
| Cr/polyimide, $h_f$ = 400 nm | Mode C | $\sigma_{\mathrm{str}}$ | 1.080 | 23 |
| DLC/Si, $h_f$ = 300 nm | three-channel | $\sigma_0$ | 1.069 | 67 |
| DLC/Si, $h_f$ = 300 nm | three-channel | $\Gamma$ | 1.068 | 64 |
| DLC/Si, $h_f$ = 600 nm | three-channel | $\sigma_0$ | 1.058 | 70 |
| DLC/Si, $h_f$ = 600 nm | three-channel | $\Gamma$ | 1.065 | 60 |
| Mo/Si, $h_f$ = 200 nm | three-channel | $\sigma_0$ | 1.115 | 47 |
| Mo/Si, $h_f$ = 200 nm | three-channel | $\Gamma$ | 1.099 | 49 |

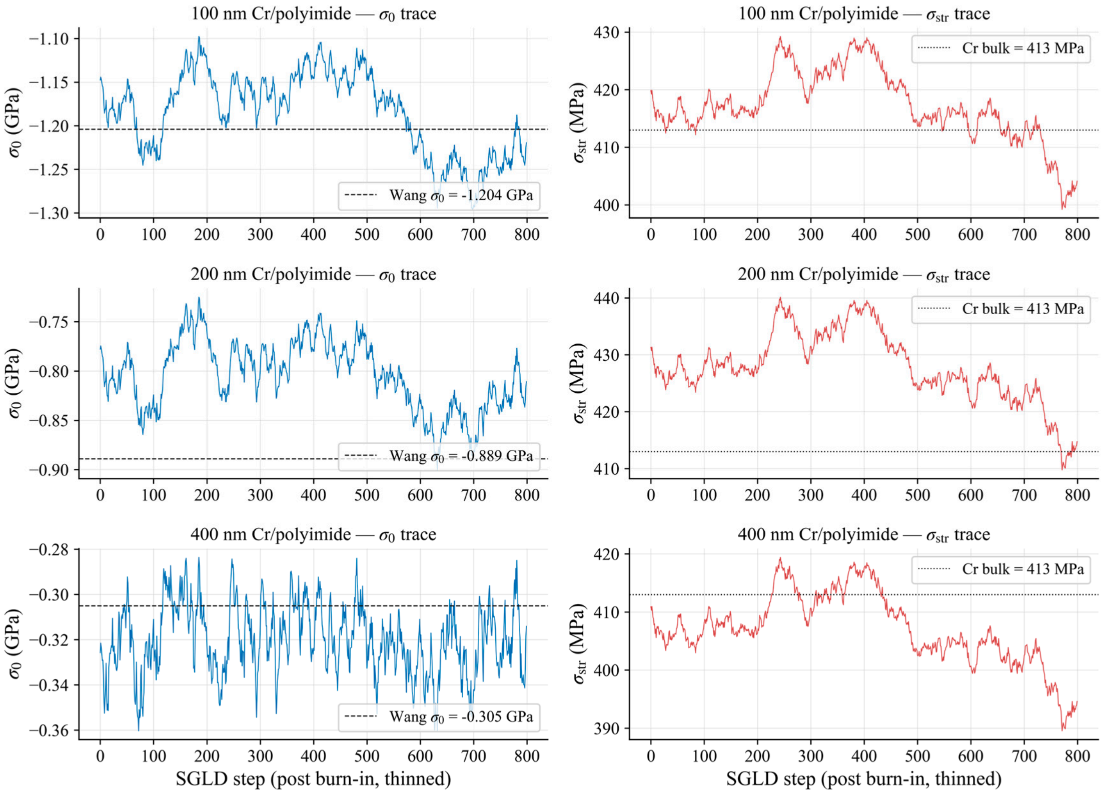


**Figure S3. Representative trace plots for the Cr/polyimide Mode C inversion.** Mixing of $\sigma_0$ (blue, left column) and $\sigma_{str}$ (red, right column) at film thicknesses of 100, 200, and 400 nm, after the 2000-step burn-in and 5-fold thinning. The dashed line marks the residual stress reported in [8], $\sigma_0$. The dotted line marks the Cr bulk fracture strength of 413 MPa. The traces fluctuate around stationary means consistent with the Mode C posterior estimates in Table 1 of the main text. Cross-chain convergence statistics for four independent chains are reported in Table S1.

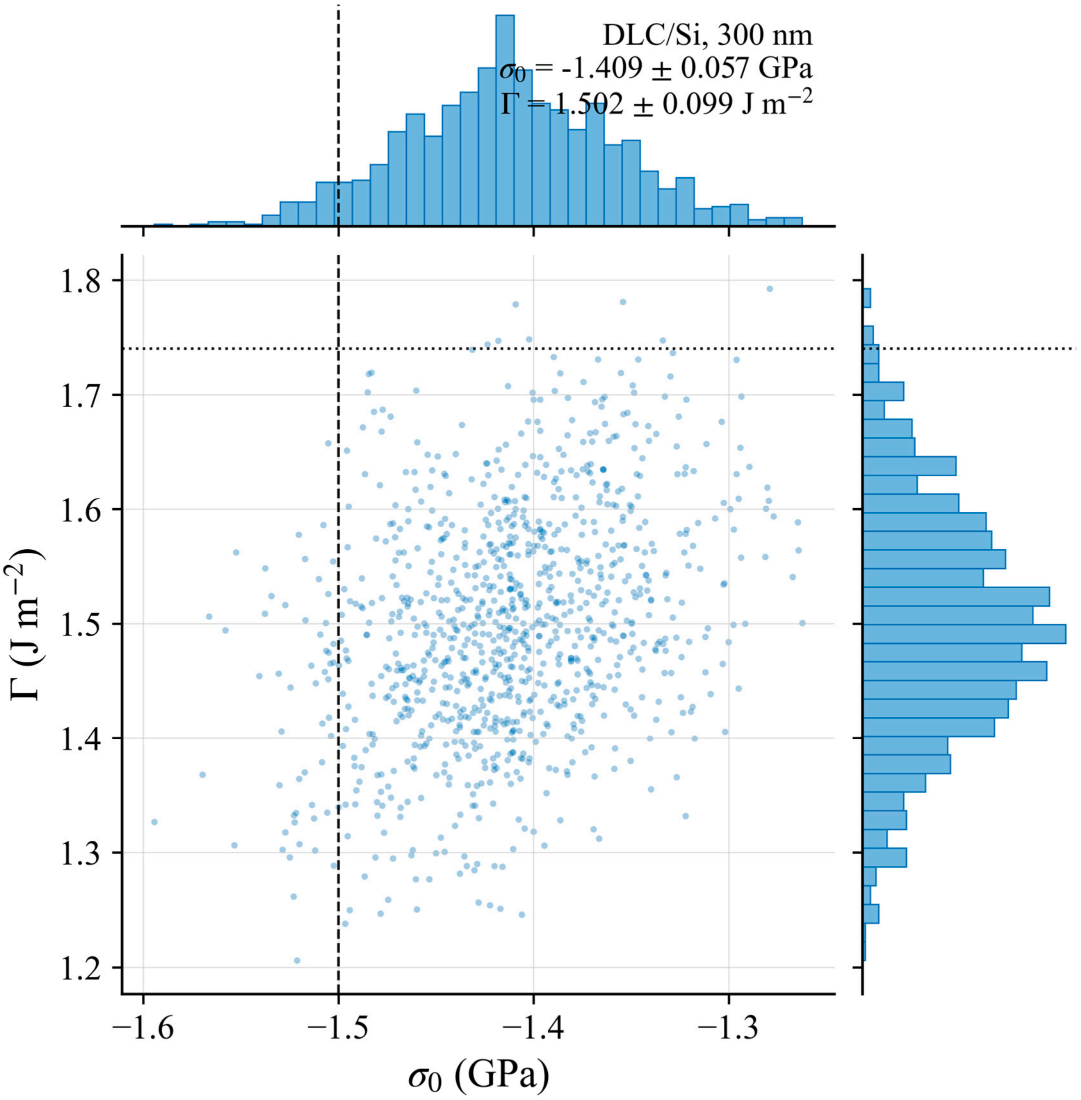
DLC/Si, 300 nm
σ0 = -1.409 ± 0.057 GPa
Γ = 1.502 ± 0.099 J m−2
Γ (J m−2)
1.8
1.7
1.6
1.5
1.4
1.3
1.2
−1.6
−1.5
−1.4
−1.3
σ0 (GPa)

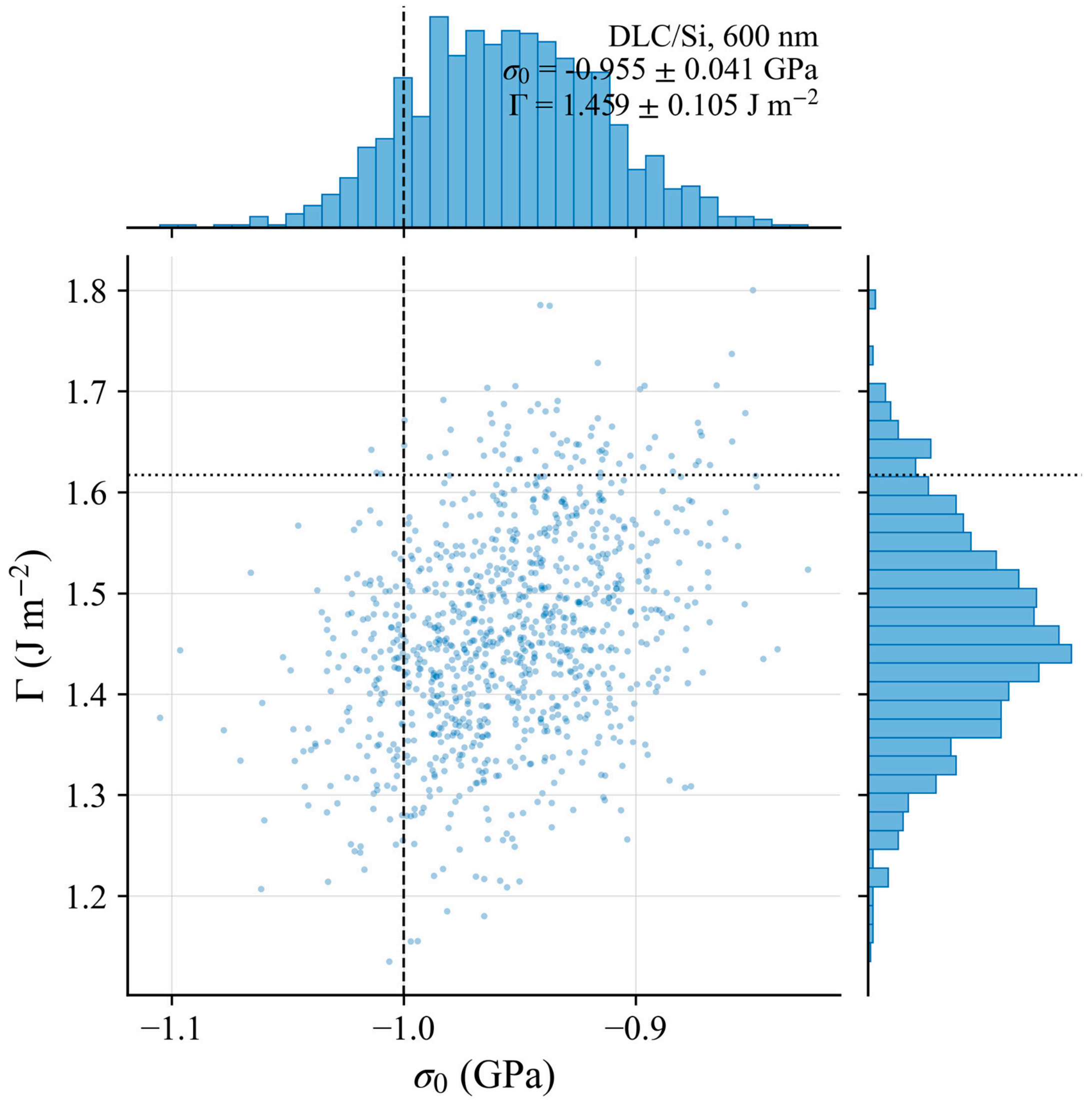


**Fig. S4 Posterior diagnostics for the DLC/Si three-channel inversion.** Top: 300 nm film. Bottom: 600 nm film. Each panel shows the joint and marginal posteriors of $\sigma_0$ and Γ under combined curvature, crack-spacing, and buckle-wavelength observations, with 5 percent independent Gaussian noise on each channel. Reference markers indicate the target values.

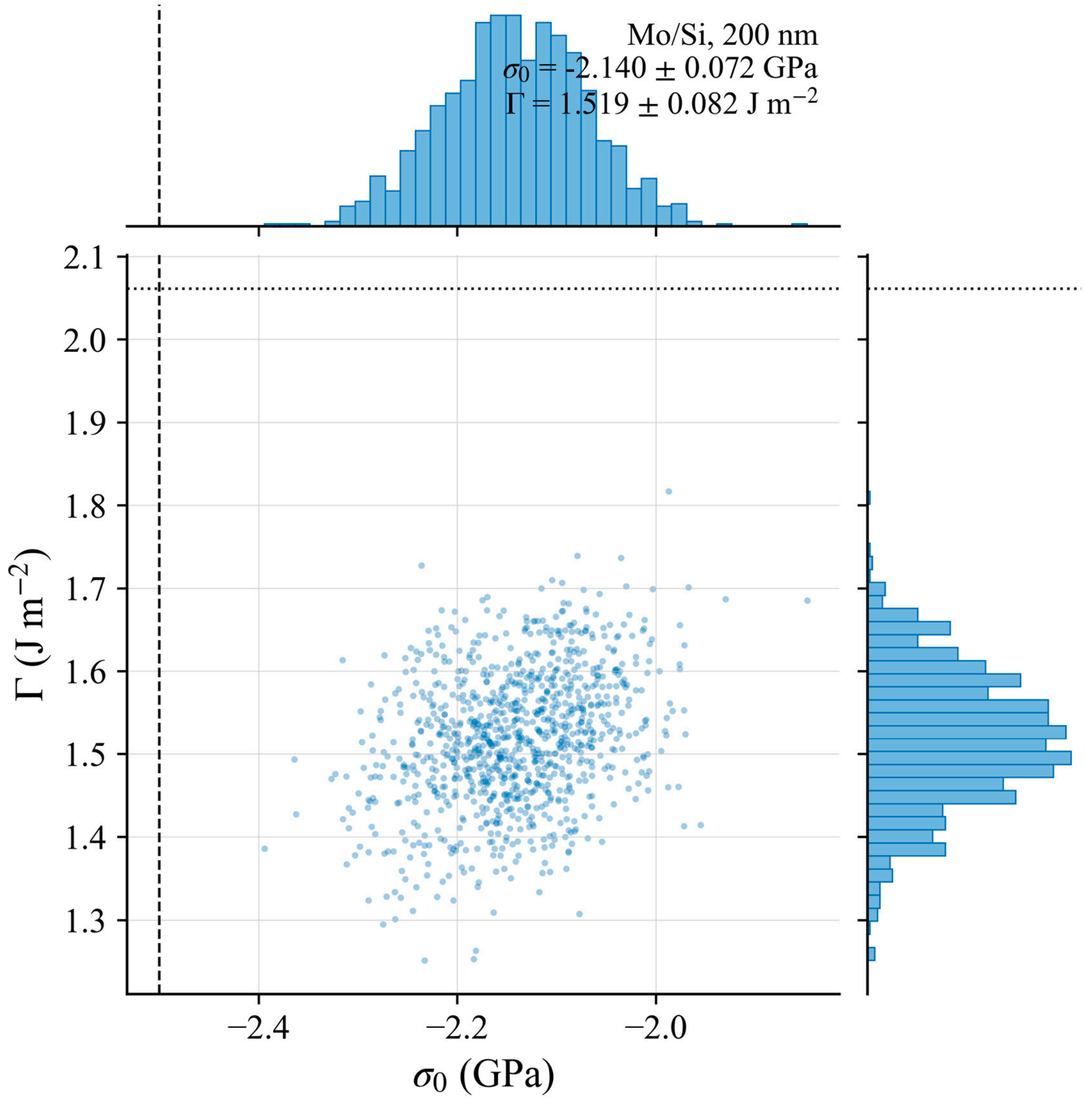


**Figure S5. Posterior diagnostics for the Mo/Si three-channel inversion.** Joint and marginal posteriors of $\sigma_0$ and $\Gamma$ for a 200 nm Mo film with target values $\sigma_0 = -2.5$ GPa and $\Gamma = 2.06$ J m$^{-2}$. The systematic low bias on $\Gamma$ is consistent with the discussion in Section 6 of the main text.

## S3. Sensitivity to the prior on the film fracture strength

The Gaussian prior on $\sigma_{str}$ in the main text is centred at the bulk value of 413 MPa with a standard deviation of 50 MPa. To probe the robustness of the inference to this choice, we repeated the Cr/polyimide Mode C inversion for three alternative prior standard deviations: 25, 50, and 100 MPa. The resulting posterior summaries are reported in Table S2.

Across the three prior widths, the posterior mean of $\sigma_0$ is stable: it changes by less than its own posterior standard deviation for every film thickness, and the 90% credible interval always contains the residual stress reported in [8]. The posterior standard deviation of $\sigma_{str}$ scales approximately linearly with the prior standard deviation. Both behaviours are consistent with the identifiability structure analysed in Section 4.2 of the main text: the curvature channel constrains $\sigma_0$ independently of the prior, while $\sigma_{str}$ remains partially informed by the prior whenever the crack-spacing curve alone does not fully determine it. The qualitative conclusions of the main text are therefore insensitive to the prior width, provided the prior is broad enough to admit departures from the bulk strength.

**Table S2. | Prior sensitivity analysis for the Cr/polyimide Mode C inversion.** Three alternative Gaussian priors on $\sigma_{\mathrm{str}}$ are considered, each centred at 413 MPa, with standard deviations of 25, 50, and 100 MPa. The 50 MPa values reproduce those reported in Table 1 of the main text.

| $h_f$ (nm) | Prior $\sigma$ (MPa) | $\sigma_0$ posterior (GPa) | $\sigma_{\mathrm{str}}$ posterior (MPa) |
|---|---|---|---|
| 100 | 25 | −1.246 ± 0.054 | 403.4 ± 6.3 |
| 100 | 50 | −1.185 ± 0.047 | 417 ± 6 |
| 100 | 100 | −1.248 ± 0.054 | 402.0 ± 6.1 |
| 200 | 25 | −0.835 ± 0.042 | 413.3 ± 6.7 |
| 200 | 50 | −0.805 ± 0.035 | 428 ± 6 |
| 200 | 100 | −0.834 ± 0.042 | 414.5 ± 6.5 |
| 400 | 25 | −0.324 ± 0.016 | 397.8 ± 6.8 |
| 400 | 50 | −0.320 ± 0.015 | 407 ± 6 |
| 400 | 100 | −0.324 ± 0.016 | 394.9 ± 6.7 |

## S4. Sensitivity to the noise level

The relative noise standard deviation $\sigma_n = 0.08$ used in the main text matches the scatter visible in Fig. 10 of [8]. To assess the impact of this choice, we repeated the Cr/polyimide Mode C inversion with $\sigma_n \in \{0.04, 0.08, 0.16\}$, spanning a factor-of-two variation about the baseline. The results are summarised in Table S3.

The posterior means of $\sigma_0$ remain stable across this range, and the 90% credible interval continues to bracket the reference value, even at twice the baseline noise. The posterior standard deviations of both parameters scale approximately linearly with $\sigma_n$, as expected for a Gaussian likelihood in the absence of strong prior tension. The qualitative findings of the main text are therefore robust to a factor-of-two misspecification of the noise level.

**Table S3. | Noise-level sensitivity analysis for the Cr/polyimide Mode C inversion.** Three alternative noise levels $\sigma_n$ are considered. The $\sigma_n = 0.08$ values reproduce those reported in Table 1 of the main text.

| $h_f$ (nm) | $\sigma_n$ | $\sigma_0$ posterior (GPa) | $\sigma_{\mathrm{str}}$ posterior (MPa) |
|---|---|---|---|
| 100 | 0.04 | −1.187 ± 0.053 | 417.2 ± 6.0 |
| 100 | 0.08 | −1.185 ± 0.047 | 417 ± 6 |
| 100 | 0.16 | −1.253 ± 0.054 | 390.7 ± 5.0 |
| 200 | 0.04 | −0.823 ± 0.042 | 425.8 ± 6.1 |
| 200 | 0.08 | −0.805 ± 0.035 | 428 ± 6 |
| 200 | 0.16 | −0.831 ± 0.041 | 401.6 ± 5.1 |
| 400 | 0.04 | −0.324 ± 0.016 | 405.6 ± 5.4 |
| 400 | 0.08 | −0.320 ± 0.015 | 407 ± 6 |
| 400 | 0.16 | −0.323 ± 0.016 | 385.6 ± 5.1 |

## S5. Forward-model implementation details

The forward map $\sigma^{f}_{x,\max}(\sigma_0, \varepsilon_{\mathrm{appl}}, \bar{L})$ defined by Eqs. (1)–(3) of the main text is implemented in PyTorch 2.x with automatic differentiation. The geometric coefficients $c_1$, $d_1$, and $d_2$ are obtained once per material system by bracketed bisection to a tolerance of $10^{-8}$. Bracket endpoints were determined by a coarse parameter sweep and verified to lie strictly on opposite sides of the corresponding root. The intervals used are listed in Table S4.

SGLD updates use PyTorch's torch.autograd.grad, and the chain state is checkpointed every 500 iterations to a CSV file. A single 6000-iteration chain (2000 burn-in plus 4000 retained) completes in approximately 3 minutes on one Intel i9-13900K core. The full

Cr/polyimide re-analysis, comprising three thicknesses, three inference modes and four parallel chains (36 chains in total), runs in approximately 2 hours on a 16-core workstation.

**Table S4.** | Bracketing intervals used in the bisection solution of the transcendental geometry equations for each material system considered in this work. The coefficients $c_1$, $d_1$, and $d_2$ are roots of the equations derived in [8] and depend only on the elastic constants and the film/substrate thickness ratio. Values for $c_1$ are obtained from $d_1$ via the algebraic relation in [8].

| System | $h_f/h_s$ | Bracket for $c_1$ | Bracket for $d_1$ (rad $m^{-1}$) | Bracket for $d_2$ (rad $m^{-1}$) |
|---|---|---|---|---|
| Cr/polyimide, 100 nm | $8.0\times10^{-4}$ | derived from $d_1$ | $(10^{-3},\ 1.49\times10^{7})$ | $(1,\ 2.51\times10^{4})$ |
| Cr/polyimide, 200 nm | $1.6\times10^{-3}$ | derived from $d_1$ | $(10^{-3},\ 7.46\times10^{6})$ | $(1,\ 2.51\times10^{4})$ |
| Cr/polyimide, 400 nm | $3.2\times10^{-3}$ | derived from $d_1$ | $(10^{-3},\ 3.73\times10^{6})$ | $(1,\ 2.51\times10^{4})$ |
| DLC/Si, 300 nm | $6.0\times10^{-4}$ | derived from $d_1$ | $(10^{-3},\ 4.97\times10^{6})$ | $(1,\ 6.28\times10^{3})$ |
| DLC/Si, 600 nm | $1.2\times10^{-3}$ | derived from $d_1$ | $(10^{-3},\ 2.49\times10^{6})$ | $(1,\ 6.28\times10^{3})$ |
| Mo/Si, 200 nm | $4.0\times10^{-4}$ | derived from $d_1$ | $(10^{-3},\ 7.46\times10^{6})$ | $(1,\ 6.28\times10^{3})$ |

## S6. Network architecture and sampler settings

The architecture of the physics-informed network and the settings of the optimiser and the posterior sampler are summarised in Table S5; they correspond to the description in Section 3.5 of the main text. The displacement network is a fully-connected multilayer perceptron with activations; the residual stress, fracture strength, and adhesion energy are carried as additional trainable scalar parameters, and the joint posterior is sampled by stochastic gradient Langevin dynamics and cross-checked with a deep ensemble and Monte Carlo dropout.

**Table S5.** | Architecture of the physics-informed network and settings of the optimiser and posterior sampler, corresponding to Section 3.5 of the main text.

| Component | Setting |
|---|---|
| Network type | fully-connected MLP (displacement field) |
| Input / hidden / output | 1 / 3 layers × 24 units / 2 |
| Activation (hidden / output) | tanh / linear |
| Trainable physical parameters | $\sigma_0$, σ_str, Γ (scalars) |
| Collocation points (PDE residual) | 64 along the film mid-plane |
| Point-estimate optimiser | Adam, lr $5\times10^{-3}$, grad-clip 5.0, 2500–3000 it |
| Refinement | L-BFGS, strong-Wolfe, ≤100–200 it |
| Noise model σ_n | 0.08 (crack spacing); 0.05 (curvature, buckle) |
| PDE-residual weight | 0.1 |
| SGLD step sizes (preconditioned) | $5\times10^{-6}$ ($\sigma_0$); $5\times10^{-6}$–$5\times10^{-5}$ (σ_str, Γ) |
| SGLD burn-in / samples / thinning | 2000–3000 / 4000–8000 / 5 |
| Deep ensemble | 10 members, independent init + noise |
| MC dropout | p = 0.05 |
| Chains for diagnostics | 4 independent |
| Precision / hardware | float64, PyTorch, CPU (minutes/thickness) |
